# The association between preoperative Mini-Cog© score and postoperative delirium (POD): a retrospective cohort study

**DOI:** 10.1186/s13741-022-00249-0

**Published:** 2022-04-21

**Authors:** S. Fiamanya, S. Ma, D. R. A. Yates

**Affiliations:** 1 Cross Lane Hospital, Tees, Esk and Wear Valley NHS Foundation Trust, Cross Lane, Scarborough, YO12 6DN UK; 2 York Hospital, York Teaching Hospitals NHS Foundation Trust, Wiggington Road, York, YO31 8HE UK; 3 Academic Alliance of Perioperative Medicine, Hull York Medical School, Heslington, UK

**Keywords:** Delirium, Screening, Cognitive impairment, Preoperative assessment, Mini-Cog©

## Abstract

**Background:**

The onset of delirium after major surgery is associated with worse in-hospital outcomes for major surgical patients. Best practice recommends assessing surgical patients for delirium risk factors and this includes screening for cognitive impairment. The Mini-Cog© is a short instrument which has been shown to predict postoperative delirium (POD) and other complications in elderly patients undergoing major elective surgery. The primary aim of this study was to ascertain whether a positive preoperative Mini-Cog© is associated with postoperative delirium in elective colorectal surgery patients at high-risk of mortality due to age or comorbidity. Secondary outcomes were 90-day mortality and length of stay.

**Methods:**

This is a retrospective analysis of data gathered prospectively between October 2015 and December 2017. Baseline data were collected at a preoperative screening clinic, and postoperative data during daily ward rounds by the Perioperative Medicine team at The York Hospital.

**Results:**

Three hundred nineteen patients were included in the final analysis, of which 52 (16%) were found to be cognitively impaired on the Mini-Cog©. Older patients (median difference 10 years, *p* < 0.001) and patients with cognitive impairment (OR 3.04, 95%CI 1.15 to 8.03, *p* = 0.019) were more likely to develop postoperative delirium in univariate analysis; however, cognitive impairment (OR 0.492, 95%CI 0.177 to 1.368, *p* = 0.174) loses its significance when controlled for by confounding factors in a logistic regression model. Cognitive impairment (OR 4.65, 95%CI 1.36 to 15.9, *p* = 0.02), frailty (OR 7.28, 95%CI 1.92 to 27.58, *p* = 0.009), American Society of Anesthesiologists (ASA) grade (OR 5.95, 95%CI 1.54 to 22.94, *p* = 0.006) and age (median difference 10 years, *p* = 0.002) were significantly associated with 90-day mortality in univariate analysis. Sex was the only factor significantly associated with length of stay in the multiple regression model, with males having a 3-day longer average length of stay than females (OR = 2.94, 95%CI 0.10–5.78).

**Conclusions:**

Mini-Cog© is not independently associated with post-operative delirium in high-risk elective colorectal surgery patients in this cohort. Mini-Cog© shows promise as a possible predictor of 90-day mortality. Larger studies exploring preoperative cognitive status and postoperative confusion and mortality could improve risk-stratification for surgery and allocation of resources to those patients at higher risk.

## Background

Delirium can be defined as an acute decline in cognitive function and attention (American Geriatrics Society Expert Panel, 2015). It is reported to be the commonest surgical complication in older adults, affecting up to 50% of older surgical patients at an annual cost of $150 billion in the USA alone (American Geriatrics Society Expert Panel, 2015). Postoperative delirium (POD) in patients who have undergone major elective surgery is associated with worse in-hospital outcomes that include longer length of stay, discharge to a higher level of care than that which was in place at admission and increased mortality (Abelha et al., 2013; Chaiwat et al., 2019; Cunningham & Kim, 2018; Korc-Grodzicki et al., 2015; Raats et al., 2015; Robinson et al., 2009b). Patients with POD have been shown to have an increased risk of mortality at 6-month follow-up (Abelha et al., 2013; Robinson et al., 2009b) as well as higher rates of hospital readmission and a decrease in overall function (American Geriatrics Society Expert Panel, 2015; Crocker et al., 2016) than patients without POD. This has led to the American Society of Anesthesiologists describing it as a public health concern and setting up the Brain Health Initiative (Perioperative Brain Health Initiative [Internet], 2020). This project campaigns to improve awareness of the importance of cognitive screening and the adoption of cognition protective measures in the perioperative period.

Preoperative assessment reviews a patient’s physiological status in order to predict perioperative outcomes; a process complicated by the complexities of surgery in elderly patients with multiple comorbidities (Foo, 2013). Systematic reviews have identified cognitive impairment as a predictor of postoperative delirium in elderly patients (Dasgupta & Dumbrell, 2006; Oh et al., 2015; Raats et al., 2016; Yang et al., 2016), and it is recommended that preoperative assessment includes a measurement of patients’ cognitive status (American Geriatrics Society Expert Panel, 2015). However, a systematic review of prediction models for delirium in older adult inpatients found them to be inadequate (Lindroth et al., 2018).

The Mini-Cog© is a short instrument which has been validated to screen for cognitive impairment in a broad range of patients (Mini-Cog©, 2018). It consists of a three-word recall test for memory, and a clock drawing test of visuo-spatial and executive function. It is scored out of five, and a score of less than three has been validated for dementia screening (Mini-Cog©, 2018). It can easily be integrated into preoperative assessment workflows (Culley et al., 2016) in 2.5 min or less (Long et al., 2012). In addition Mini-Cog© appears to predict POD in elderly patients undergoing elective surgery (Culley et al., 2017; Dworkin et al., 2016; Robinson et al., 2012), with the proportion of patients developing delirium increasing by as much as nine times in those with low versus high scores (Dworkin et al., 2016). Preoperative Mini-Cog© score has been shown to have a negative predictive value (NPV) for delirium of up to 94%, indicating its potential as a screening tool to identify those at low risk of postoperative delirium (Dworkin et al., 2016). Mini-Cog© has also been shown to be associated with postoperative complications other than delirium in older surgical patients, including odds of being discharged to a place other than home, risk of longer hospital length of stay (Culley et al., 2017) and 6-month mortality (Robinson et al., 2009a; Robinson et al., 2012). Most studies have primarily investigated the relationship between Mini-Cog© and POD using univariate (Dworkin et al., 2016; Robinson et al., 2012) or age-adjusted analysis (Culley et al., 2017). When more comprehensive, multivariate analyses have been used, links between Mini-Cog© and POD have been variable, with some studies showing a continued effect (Dworkin et al., 2016; Robinson et al., 2009b), and others not (Robinson et al., 2012).

## Methods

### Aim

The aim of this observational study was to identify the relationship between preoperative Mini-Cog© scores and POD in patients undergoing elective colorectal surgery when controlling for other physiological and demographic variables that could possibly influence the incidence of POD. Secondary outcome measures were the relationship between Mini-Cog© scores and 90-day mortality and length of stay.

### Data collection

This is a retrospective analysis of prospectively collected data routinely gathered between October 2015 and December 2017 at The York Hospital. Data were analysed for patients older than 55 years (or over 50 years if they had significant comorbidities) undergoing major elective colorectal surgery. This includes routine collection of perioperative risk factors and outcomes for local audit and service development. As all data are collected routinely and anonymised, the York & Scarborough Teaching Hospitals NHS Foundation Trust Research and Development department viewed this project as ‘low risk research’ and as such no formal ethics committee review was required. The Health Research Authority confirmed this and HRA approval was obtained (IRAS Project ID 243694).

Patients that did not undergo planned surgery or had no Mini-Cog© assessment were excluded. Preoperative variables that were thought to be possible factors in influencing POD included ASA grade, Rockwood Clinical Frailty Scale score (frailty score) and a determination of anaerobic threshold (AT) via cardiopulmonary exercise testing. Outcome data were collected prospectively by a Perioperative Medicine Nurse Specialist (SM), and confusion was assessed during a daily clinical review. Delirium was defined by clinically significant new confusion, fluctuating attention, or impaired cognition. Outcome data for 90-day mortality and hospital length of stay were collected after patient discharge from the Trust’s electronic database.

### Statistical analysis

A previous study by Colley et al. demonstrated incidences of POD in a cohort of elderly orthopaedic patients, of 21% in those with poor MiniCog© scores (< 3) versus 7% in those who scored ≥ 3 (Culley et al., 2017). To investigate a similar level of preoperative cognitive impairment and POD in our surgical cohort would require 300 patients (assuming α=0.05 and a power of 0.9). This sample size would also allow exploratory analysis of other variables that may influence POD in multivariate analysis.

All data were anonymised and held on a secure MS Access database, then exported via Microsoft Excel to SPSS version 19.0 for analysis. A Mini-Cog© score < 3 was used as the cut-off for cognitive impairment. Variables were selected based on their clinical significance. ASA grade was dichotomised into low (< 3) and high (≥ 3) and frailty score was dichotomised into not-frail (< 4) and frail (≥ 4) in order to ensure significant events for robust analysis due to highly skewed distributions, and to enable a clinically relevant interpretation. Baseline characteristics of preoperatively low scoring Mini-Cog© vs normal scoring Mini-Cog© patients were first tested for distribution, then compared using Mann-Whitney *U* tests for non-parametric continuous data and chi-squared tests for categorical data. Associations between Mini-Cog© score and POD were investigated by univariate analysis using Mann-Whitney *U* or Fisher’s exact tests for continuous data and chi-squared tests for categorical data. For variables associated with the outcome and Mini-Cog© score at an alpha level ≥ 0.1, binary logistic regression was used to determine if Mini-Cog© score was independently associated with POD. Associations between Mini-Cog© score and 90-day mortality were investigated by univariate analysis using Mann-Whitney *U* or Fisher’s exact tests for continuous data and chi-squared tests for categorical data. Associations between Mini-Cog© score and length of stay were investigated by multiple linear regression analysis. Associations were deemed significant at *p* < 0.05.

## Results

### Baseline characteristics

Data were collected for 357 perioperative patients, with 319 included in the final analysis and 36 excluded due to incomplete data or not meeting inclusion criteria. Fifty-two (16%) were found to be cognitively impaired. Patients with cognitive impairment were older than unimpaired patients (75 years [IQR 11] vs 70 years [IQR 13], *p* = 0.001). They were also twice as likely to have an ASA grade ≥ 3 (OR 2.14, 95%CI 1.17 to 3.91, *p* = 0.012). Frailty was recoded into a dichotomous variable. Only two patients (0.6%) were recorded as having a formal dementia diagnosis. This likely reflects underdiagnosis rather than the true prevalence and consequently dementia was excluded as an independent variable. Baseline characteristics are shown in Table 1.

**Table 1:** Baseline characteristics

	**Mini-Cog© Score**		
	**Low**	**Normal**	
**Participants**	52 (16%)	267 (84%)	
**Age** (years) *median (IQR)*	75 (11)	70 (13)	*p* = 0.001*
**Frailty score** *median (IQR)*	2 (1)	2 (1)	*p* = 0.069*
**Anaerobic threshold** (ml/kg/min) *(median (IQR)*	11.0 (4)	11.9 (4)	*p* = 0.153*
**Sex** *n (%)*			
Female	29 (56%)	107 (40%)	*p* = 0.36**
Male	23 (44%)	160 (60%)	
**ASA grade**			
< 3	27 (52%)	185 (70%)	p = 0.012**
> = 3	25 (48%)	80 (30%)	
**Surgery** *n (%)*			
Colonic	31 (60%)	148 (55%)	
Rectal	21 (40%)	116 (43%)	
Mixed/other	0 (0%)	3 (1%)	

*ASA* American Society of Anesthesiologists grade, *IQR* interquartile range

*Mann-Whitney *U*

**Chi-squared test

### Postoperative delirium

Overall incidence of POD was 6.3%. Mini-Cog© had a sensitivity of 35% and 85% specificity for predicting subsequent POD. Negative predictive value was 95%. Univariate and logistic regression analysis are shown in Table 2. Only age (OR 0.492, 95%CI 0.177 to 1.368, *p* = 0.174) remains an independent risk factor for the development of POD when controlling for other preoperative variables using binary logistic regression.

**Table 2:** Postoperative delirium

	**Delirium**	**No delirium**				
**Number**	20 (6.3%)	299 (93.7%)				
			**Unadjusted**		**Adjusted**	
	*Median (IQR)*		*Median difference*	*p*	*OR (95% CI)*	*p*^†^
**Age**	81 (9)	71 (14)	10	< 0.001*	1.13 (1.06 to 1.22)	***0.001***
**Anaerobic threshold**	11.2 (2)	11.9 (4)	0.7	0.299*		
	*n (%)*		*OR (95% CI)*	*p*		
**Sex**						
Female	11 (55%)	125 (42%)	1.7 (0.69 to 4.22)	0.25**		
Male	9 (45%)	174 (58%)				
**ASA grade**						
< 3	10 (50%)	202 (68%)	2.13 (0.86 to 5.28)	0.098**	0.82 (0.31 to 2.14)	0.69
≥ 3	10 (50%)	95 (32%)				
**Frailty**						
Frail	3 (18%)	26 (9%)	2.18 (0.59 to 8.07)	0.209***		
Not frail	14 (82%)	264 (91%)				
**Mini-Cog© score**						
Low	7 (35%)	45 (15%)	3.04 (1.15 to 8.03)	0.019**	0.49 (0.18 to 1.37)	0.17
Normal	13 (65%)	254 (85%)				
**Type of surgery**						
Laparoscopy	3 (15%)	95 (32%)		0.29**		
Open	16 (80%)	193 (65%)				
Converted	1 (5%)^	11 (4%)				

*CI* = confidence interval, *IQR* = interquartile range, *OR* = odds ratio. Bold = *p* < 0.1, bold + italics = *p* < 0.05

*Mann-Whitney *U*

**Chi-squared test

***Fisher’s exact test

^Expected cell count 0.75

†Logistic regression

### 90-day mortality

MiniCog**©** score, frailty, ASA grade and age were associated with 90-day mortality in univariate analysis (OR 4.65, 95%CI 1.36 to 15.9, *p* = 0.02) (Table 3). There were insufficient outcomes to perform logistic regression with other independent variables.

**Table 3:** Multiple linear regression for Mini-Cog© score and length of stay

	**OR**	**95% CI**		***p***
		**Lower**	**Upper**	
**Constant**	0.51	− 12.71	13.73	0.94
**Sex**	2.94	0.10	5.77	0.04
**Age**	0.15	− 0.01	0.31	0.07
**Frailty**	1.11	− 4.14	6.37	0.68
**Mini-Cog© score**	− 3.16	− 6.99	0.66	0.11
**Surgery**				
Open	2.11	− 0.81	5.04	0.16
Converted	− 1.39	− 8.97	6.19	0.72
**ASA grade**	0.84	− 2.34	4.02	0.60
**Anaerobic threshold**	− 0.34	− 0.84	0.16	0.18

*CI =* confidence interval

### Length of *s*tay

The model to predict length of stay based on age, frailty, sex, cognitive impairment, anaerobic threshold, type of surgery (open vs closed vs converted) and ASA grade was significant using ANOVA at *p* = 0.040 (*F* = 2.054, df = 8). *R*^2^ = 0.029. In the full multiple regression model, sex remained the only factor significantly associated with length of stay, with males having a 3-day longer average length of stay than females (OR = 2.94, 95%CI 0.10 to 5.78, *p* = 0.04) (Table 3). There was a high degree of skew in the normality plot of standardised residuals, and uniform variance of standardised residuals was not demonstrated. Durbin-Watson statistic was 0.794 indicating some correlation between independent variables. Tolerance statistics were above 0.2 and VIF statistics less than 2 for all variables indicating there was no multicollinearity.

## Discussion

From the results of our analyses, Mini-Cog© is not independently associated with POD or length of stay in patients undergoing elective major colorectal surgery. Whilst the Mini-Cog© is associated with POD on univariate analysis, this association is lost when other variables which are also plausibly involved in the mediation of POD are added into the logistic regression model. This is important as it highlights the likely multifactorial nature of how POD arises and the necessity to provide a comprehensive, holistic preassessment of the high-risk patient.

The Mini-Cog© was also associated with 90-day mortality in univariate analysis, although we were unable to explore this association with multiple regression due to low mortality rates. Our results are both in keeping with, and build upon, previous literature by highlighting the difficulty in predicting in-hospital delirium (Lindroth et al., 2018) and demonstrating that the link between preoperative cognition and POD may be mediated through other physiological variables (Culley et al., 2017; Dworkin et al., 2016; Robinson et al., 2012). Analysis showed that at baseline, patients with poor Mini-Cog© scores were on average 10 years older (*p* = 0.001) and had a higher ASA grade (*p* = 0.012), which may reflect a link between multimorbidity and cognitive function which is controlled for in multivariate analysis.

In our sample, 16% of patients were found to have cognitive impairment as assessed by a Mini-Cog© score < 3, and 6.3% of patients were confused postoperatively. This is at the low end for incidence of POD found in systematic reviews, which ranges from 5 to 52% (Dasgupta & Dumbrell, 2006; Raats et al., 2016; Yang et al., 2016). However, most of these studies have involved orthopaedic (mostly hip-fracture) and major vascular surgery patients. Due to the emergent and traumatic nature of clinical presentation, they would likely have higher levels of haemodynamic and general physiological instability, with poorer perioperative optimisation than the elective patients in our sample, which may explain the higher rates of POD.

We chose a Mini-Cog© cut-off of < 3, which is validated for dementia screening. For broader preoperative confusion assessment that does not reflect cognitive impairment reaching the requirements for dementia, a more generous cut-off of < 4 has been used, with relationships still found between Mini-Cog© score and POD, length of stay and even mortality (Robinson et al., 2009a; Robinson et al., 2012). Dworkin et al. showed that raising the threshold from a score < 3 to a score < 5 only slightly increased NPV from 90 to 94%, indicating minimal benefit to a higher Mini-Cog© score in the diagnosis of preoperative cognitive stability (Dworkin et al., 2016). Indeed, our NPV was 95%, further justifying our choice of 3 as the cut-off value. Our more stringent cut-off is appropriate for investigating the further role of Mini-Cog© in identifying the risk of subsequent POD.

Only two of our patients had a formal dementia diagnosis. This likely reflects underdiagnosis in our surgical population and is in keeping with a previous study that also identified no dementia diagnoses in their elderly patients (Dworkin et al., 2016). Screening for dementia via Mini-Cog©, and using the cut-off of < 3 to do so, may still be an important tool for realising long term benefits in terms of patient cognition and surgical outcomes. Possible dementia identified at a comprehensive preoperative assessment could be passed on to family doctors or specialist memory clinics either preoperatively or postoperatively, to identify those with true dementia and ensure long-term optimisation of patients.

Mini-Cog© score was associated with 90-day mortality in the unadjusted analysis (OR 4.65, 95%CI 1.36 to 15.9, *p* = 0.02). This finding is in keeping with previous evidence identifying a role for Mini-Cog© in postoperative mortality in high-risk surgical patients (Robinson et al., 2009a; Robinson et al., 2012). This could be due to poor cognition reflecting a lack in other physiological reserve, or maybe due to confused patients being more at risk of other morbidities such as falls. It is possible that poor cognition could affect patients’ ability to engage in postoperative rehabilitation techniques which act to minimise risk of developing chest infections and ileus, whilst ensuring safe mobilisation. It is unsurprising that the other physiological variables (frailty, ASA grade and age) were also associated with this, but it is not clear if Mini-Cog© score acts independently in this regard as our data did not allow us to perform a multivariate logistic regression.

Delirium is thought to be preventable in up to 40% of patients (American Geriatrics Society Expert Panel, 2015). Identification of at-risk patients would allow clinical teams to institute measures to ameliorate the effect of anaesthesia and surgery on cognition. Screening with Mini-Cog© is very quick and cheap, and whilst we have demonstrated that it does not act independently of other variables, its use could increase the awareness of clinical teams to the likelihood of patients developing POD. Increased awareness could then lead to early diagnosis via targeted screening for cognitive change using a validated screening tool such as the Confusion Assessment Method (CAM). Intraoperative measures to reduce the incidence of POD may include the use of processed electroencephalographic (e.g. bispectral index) monitoring by anaesthetists, as a lighter depth of anaesthesia has been shown to reduce rates of POD. Pharmacological management could be targeted, for example by preferentially using regional blocks in high-risk patients to minimise the use of opioid painkillers, polypharmacy and pain-induced delirium. Non-pharmacological approaches such as mobility enhancement, cognitive orientation, sleep enhancement measures and targeted nutrition and fluid therapy have also been demonstrated to be effective (American Geriatrics Society Expert Panel, 2015).

## Limitations

Our cohort contained very few patients with a formal diagnosis of dementia and this prevented us from including this important and relevant diagnosis as a variable in our analysis. It is possible that this reflects incomplete coding of the patient group rather than very low rates of dementia. The study may also be underpowered given the low incidence of POD in our sample compared to other studies.

It is clear from our analysis that the association between the Mini-Cog© score and POD is influenced by other factors. We collected data on some of those factors but there will be other variables (surgical magnitude, duration of anaesthesia/surgery, blood loss, etc.) at play within our specific cohort of colorectal patients which we did not record and analyse. This does limit the external validity and therefore generalisability of our analysis. Further exploratory work investigating other perioperative factors in wider surgical groups is warranted.

A further limitation is the accurate capture of the outcome of POD in patients. This is judged clinically during postoperative ward rounds and is subject to possible inter-rater variability, particularly in missing cases of hypoactive delirium. In a study comparing relationships between Mini-Cog© using delirium assessed using the CAM with delirium assessed by review of clinical notes, age-adjusted OR remained of similar magnitude and statistical significance (CAM OR 4.52, 95%CI 1.3 to 15.68 *p* = 0.017 vs. notes OR 3.41, 95%CI 1.26 to 9.23, *p* = 0.016). It is therefore possible this may have affected our analysis; however, it is unlikely this would be significant enough to alter the interpretation. Nevertheless, future studies could be improved by formally screening for delirium in daily ward rounds using a tool such as the CAM.

## Conclusion and recommendations

Mini-Cog© is not independently associated with POD in high-risk colorectal surgery patients. It is associated with 90-day mortality in univariate analysis. Further exploration is warranted to confirm its role in relation to other physiological variables. These relationships should be investigated in further retrospective or prospective analyses using a larger sample sizes, and in studies investigating the causes of mortality specifically in postoperatively confused patients. Importantly, this study builds on the body of literature that argues a holistic assessment of presurgical morbidity which includes brain end-organ dysfunction is essential, and provides evidence that pre-existing physiological criteria may be better suited to calculate this.

## Data Availability

The datasets used during the current study are available from the corresponding author on reasonable request.

## Abbreviations

ANOVA: Analysis of variance
ASA: American Association of Anesthesiologists
AT: Anaerobic threshold
BIS: Bispectral Index™
CI: Confidence interval
CAM: Confusion Assessment Method
CAM-ICU: Confusion Assessment Method for the Intensive Care Unit
df: Degrees of freedom
IQR: Interquartile range
OR: Odds ratio
NPV: Negative predictive value
POD: Postoperative delirium
VIF: Variance inflation factor
